# An Unusual Presentation of Complex Regional Pain Syndrome Type 1

**DOI:** 10.7759/cureus.46333

**Published:** 2023-10-01

**Authors:** Abdullah A Alghamdi, Sawsan F Almalki, Alanoud A AlRakban, Shahad E Alshail, Hadeel F Almajid, Norah A Bin Sulaiman

**Affiliations:** 1 Anesthesiology and Pain Medicine, Johns Hopkins Aramco Healthcare, Dammam, SAU; 2 Medicine and Surgery, Alfaisal University College of Medicine, Riyadh, SAU

**Keywords:** analgesia, multidisciplinary, anesthesia, crps, complex regional pain syndrome

## Abstract

Complex regional pain syndrome (CRPS) is a chronic neurologic painful disorder usually present after a traumatic insult. It is divided into two subtypes based on the absence of a significant nerve injury: type 1 or dystrophy and type 2 or causalgia. The exact mechanism still needs to be fully understood. The management of CRPS requires a multidisciplinary team approach with a rehabilitation program and physical and occupational therapies.

We present a case report of a 22-year-old Saudi female with no medical or surgical history who presented to the clinic with severe pain, swelling, and discoloration in the left lower limb associated with unusual symptoms of non-epileptic convulsion attack and short-term memory loss for three years that increased in intensity. There was marked swelling and discoloration of the left lower limb, which was more significant at the foot, and the limb was tender and warm to the touch and allodynia. A slight touch to the limb led to a whole-body non-epileptic convulsion lasting for less than 30 seconds and loss of short-term memory and consciousness following the convulsion attack. A multidisciplinary team primarily managed the patient.

In this case, the rarity and refractory to medical management emphasize the importance of understanding the different therapeutic modalities in managing this syndrome. However, more studies are warranted to understand the exact cause, pathogenesis, and available treatment options.

## Introduction

Complex regional pain syndrome (CRPS) is a chronic neurologic pain disorder that usually presents after a traumatic insult [1,2]. Annually, 5.4-6.2 per 100,000 people are affected [2]. CRPS is divided into two subtypes based on the absence of a significant nerve injury: type 1 or dystrophy and type 2 or causalgia [2]. It is a female-dominant disorder with risk factors that include an upper extremity injury and high-energy trauma [2]. The exact mechanism still needs to be fully understood. Therefore, a better understanding of the nature, degree, and related conditions is needed to improve the management and treatment of CRPS [1]. Skin sensitivity, limb-restricted pain, swelling, color and temperature changes, inability to initiate and control movement, and feelings of disconnection from the affected limb are all typical symptoms of CRPS [1]. It is a monophasic syndrome that is diagnosed clinically by the presence of these symptoms, which affect a patient’s quality of life and daily activities and require attention to overcome unpleasant long-term outcomes [1].

The management of CRPS requires a multidisciplinary team approach with a rehabilitation program and physical and occupational therapies [3]. As it is associated with severe pain, referral to a pain management clinic is crucial for analgesia and other options, such as oral corticosteroids, anticonvulsants, and analgesic antidepressants [3].

## Case presentation

**Figure 1:**
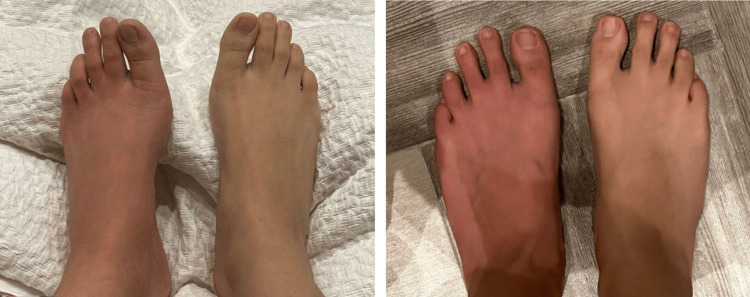
Persistent marked swelling and discoloration of the left foot despite being previously treated with radiofrequency ablation of lumbar sympathetic nerves

A 22-year-old Saudi female with no medical or surgical history presented to the clinic with severe pain, swelling, and discoloration in the left lower limb with unusual symptoms of non-epileptic convulsions and short-term memory loss that increased in intensity over three years. The pain, which was rated a 10 on the 1-10 pain scale, was continuous and aggravated by movement and touch. There was no history of trauma. However, invasive procedures were reported; lumbar sympathetic nerve blocks and radiofrequency ablations were performed multiple times throughout the three years. Upon physical examination, there was marked swelling and discoloration of the left lower limb, which was more significant at the foot (Figure 1).

The limb was tender and warm to the touch. A slight touch to the limb led to a generalized non-epileptic convulsion lasting for less than 30 seconds and post-ictal loss of memory and consciousness. There was no sensory or motor loss with a normal range of motion, both passive and active. The patient reported difficulties performing daily activities because of the severity of the pain and its associated symptoms. The rest of the examination was unremarkable.

The results of further investigation such as MRI and EEG rule out seizures, and laboratory workups were all within normal limits, with no elevation in inflammatory markers. A bone scan with technetium-99m (Tc-99m) demonstrated a mild increase in tissue perfusion, vascularity, and periarticular tracer uptake in the left foot and ankle, mainly in the left tarsal and metatarsal joints and the left calcaneus, without corresponding osseous pathology. The remainder of the skeleton demonstrated normal findings.

As initial pain management, the patient was managed with multiple analgesics, such as one tablet oral acetaminophen 325 mg, IV morphine 1 mg/mL, IV ketamine 25 mg, oral pregabalin 75 mg, IV fentanyl 500 mcg, and oral tramadol 50 mg. A detailed discussion about inserting a spinal cord stimulator followed. After the spinal cord stimulator operation, the patient reported noticeable relief, rated as 7 out of 10 on the relief scale, and the disappearance of the limb’s symptoms of swelling, pain, discoloration, and convulsions. The patient tolerated the procedure well, with no immediate or late complications. She was discharged safely after three days with home medications, analgesia to be taken as needed (PRN), and a clinic follow-up.

After two months, the patient was admitted for the relapse of all previous symptoms in the lower limbs with a new presentation affecting her left hand. The pain was primarily managed with a sympathetic ganglion nerve block. Further pain management included prescribed Lyrica (pregabalin) 300 mg daily, lidocaine patches (5%), and sympathetic ganglion nerve blocks from time to time. Overall, the patient’s condition improved tremendously, demonstrated by her ability to perform daily activities with no significant pain.

## Discussion

CRPS is a life-altering condition that usually affects the extremities after a trauma or nerve injury, leading to psychological comorbidities, such as isolation and depression [2]. Several systematic reviews have been conducted to highlight the incidence and pathological mechanisms of developing this syndrome. Few articles have examined CRPS; hence, establishing and publishing research on this syndrome is crucial to providing clear evidence for managing and treating these patients (Table 1) [4].

**Table 1:** Recent articles on CRPS pathology, disease courses, treatment, and management plans. CRPS: complex regional pain syndrome, SCS: spinal cord stimulator.

Year	Country	Authors	Study design	Objectives	Results
2022	UK	Johnson et al. [1]	Systematic review	To summarize the published data on measures of function and impact, including occupational parameters, of CRPS at 12 months from symptom onset and beyond	The ongoing impact of one episode of CRPS on limb function and work status is high [1].
2021	Canada	Mesaroli et al. [5]	Systematic review	To identify screening and diagnostic tools for CRPS and summarize their feasibility, measurement properties, and study quality, and to identify screening and diagnostic tools for CRPS in pediatric populations (0-21 years of age)	For adults with CRPS, the Budapest criteria should be used by researchers and clinicians for diagnosis in combination with clinical judgment. For pediatric CRPS, there are no valid diagnostic criteria, and caution should be taken if applying the criteria; a clinical diagnosis by a pediatric pain specialist is preferred [5].
2022	Denmark	Kunwald et al. [6]	Retrospective	To describe the effects of SCS for CRPS with known nerve injury on pain reduction and opioid use	Only half of the patients experienced a clinically significant response, and the costs and complications associated with SCS were considerable [6].
2021	Italy	Taylor et al. [7]	Comprehensive review	To outline better prevention, diagnosis, and treatment of CRPS	Larger and higher-quality clinical studies are needed to enable the development of precisely targeted therapies [7].

Systematic reviews conducted in the UK and Canada on the course and impact of CRPS and self-reported physical examination, respectively, highlight the significant effect of the syndrome on work status and quality of life for patients and the lack of screening tools. However, the Veldman criteria, the International Association for the Study of Pain criteria, the Budapest criteria (the recommended diagnostic tool), and an unvalidated instrument for pediatric CRPS are cited as diagnostic tools [5].

Spinal cord stimulation (SCS) to treat CRPS type 2 has been intently studied, including patients who met the Budapest criteria (2003) for this subtype and completed six- and 12-month follow-ups. Results indicate that SCS may offer clinically relevant pain reduction in CRPS type 2. However, in a cohort of 26, only half of the patients experienced a clinically significant response, and the costs and complications associated with SCS were considerable [7].

The development of CRPS is significantly influenced by neuropathic inflammation, especially by the activation of peripheral nociceptors of C-fibers. Moreover, a history of depression or post-traumatic stress disorder (PTSD) is correlated with a CRPS diagnosis. Physical therapy, medication, and interventional methods are all examples of treatment modalities. Because CRPS is multifaceted, further study and research are needed to better understand its pathophysiology, epidemiology, genetic involvement, psychological consequences, and treatments [8].

A case reported in Saudi Arabia in 2020 of a 20-year-old female with CRPS type 1 who experienced sequential spread to all four limbs despite different treatment modalities, including medical therapy, nerve block, radiofrequency ablation, and surgical sympathectomy, illustrates the need for a multidisciplinary approach to manage CRPS. The refractory disease may respond to intrathecal baclofen with morphine [9]. Findings of a repeatable pathogenic etiology for CRPS have been reported in the literature. There is scant evidence to suggest that CRPS is an autoimmune disorder, despite some assertions to the contrary. Notably, the syndrome should be handled as a subset of functional pain disorders (FNDs) and not be referred to as “CRPS” [10].

## Conclusions

This case highlights an unusual presentation of CRPS that affected the limbs and was associated with convulsive episodes followed by short-term memory loss. The rarity and resistance to medical management emphasize the importance of understanding the different therapeutic modalities in managing this syndrome. However, more studies are warranted to understand the exact cause, pathogenesis, and treatment options.
